# Loss of Stat6 affects chromatin condensation in intestinal epithelial cells causing diverse outcome in murine models of inflammation-associated and sporadic colon carcinogenesis

**DOI:** 10.1038/s41388-018-0551-2

**Published:** 2018-10-23

**Authors:** Tiago De Oliveira, Mallika Ramakrishnan, Michaela A. Diamanti, Paul K. Ziegler, Frank Brombacher, Florian R. Greten

**Affiliations:** 1 Institute for Tumor Biology and Experimental Therapy, Georg-Speyer-Haus, 60596 Frankfurt, Germany; 2 International Centre for Genetic Engineering and Biotechnology and University of Cape Town and South African Medical Research Center external Unit, Anzio Road, Observatory, 7925 Cape Town, South Africa; 3 German Cancer Consortium (DKTK) and German Cancer Research Center (DKFZ), 69120 Heidelberg, Germany

**Keywords:** Colorectal cancer, Apoptosis

## Abstract

While great advances have been achieved regarding the genetic basis of colorectal cancer, the complex role of cell–cell communication and cytokine-induced signaling during its pathogenesis remains less understood. Signal transducer and activator of transcription 6 (Stat6) is the main transcription factor of interleukin-4 (IL-4) signaling and its participation in the development of various tumor types has been already reported. Here we aimed to examine the contribution of Stat6 in intestinal epithelial cells (IEC) in mouse models of intestinal carcinogenesis. Wild-type (WT), Stat6 knockout (*Stat6*^−/−^), and intestinal epithelial cell-specific IL-4Rα knockout (*Il-4rα*^*Δ*IEC^) mice were subjected to colitis-associated (AOM/DSS) and colitis-independent (sporadic) carcinogenesis. IEC proliferation, apoptosis and RNA expression were evaluated by immunohistochemical, immunoblot, and RT-PCR analysis. We found that *Stat6*^−/−^ mice developed more tumors in the colitis-associated carcinogenesis model. This was accompanied by a more pronounced inflammatory response during colitis and an elevated Stat3-dependent proliferation of IEC. Increased sensitivity to DSS-induced colitis was caused by elevated cell death in response to the initial carcinogen exposure as *Stat6* deficiency led to increased chromatin compaction affecting DNA damage response in IEC upon treatment with alkylating agents independently of IL-4Rα engagement. Thus, loss of *Stat6* caused more severe colitis and increased tumor load, however loss-of-initiated *Stat6*^−/−^ IEC prevented tumor formation in the absence of overt inflammation. Our data unravel unexpected IL-4-independent functions of Stat6 in chromatin compaction in intestinal epithelial cells ultimately providing both tumor suppressive as well as tumor promoting effects in different models of intestinal tumorigenesis.

## Introduction

In spite of the remarkable clinical advances in cancer diagnosis and treatment achieved in the last decades, colorectal cancer continues to be an important cause of mortality worldwide with ~700,000 deaths reported yearly [1]. Thus, an exact understanding of the molecular mechanisms involved in its pathogenesis is crucial for the improvement of new therapeutic strategies. Deciphering the mutational landscape that controls oncogenic signaling in tumor cells is one pre-requisite for this. However, signaling induced by the tumor microenvironment controlling cell fate decisions as well as polarization of inflammatory immune cells also play an equally important role during all stages of tumorigenesis [2]. Depending on the prevailing polarization of T cells (T_H_1, T_H_2, T_H_17, etc.), macrophages (M1 and M2) and neutrophils (N1 and N2) the tumor microenvironment can provide both tumor promoting or tumor suppressive signals [3]. Importantly, the decision whether the microenvironment is shifted toward a more tumor suppressive or tumor promoting milieu depends on cytokine-induced signaling. One of the most prominent signaling pathways responsible to skew immune cells towards a presumably tumor promoting type 2 profile comprises IL-4 receptor engagement and subsequent activation of signal transducer and activator of transcription 6 (STAT6) [4]. IL-4 as well as IL-13 use the common γ-chain (γc)-related IL-4 receptor α-chain (IL-4Rα) to signal through three cytokine–receptor combinations: IL-4 signals through the type I receptor IL-4Rα–γc, and both IL-4 and IL-13 can signal through the type II receptor IL-4Rα–IL-13Rα1 [5]. Expression of the type I receptor is restricted to immune cells while the type II receptor is also found in other cell types, including epithelial cells [5–10]. Both IL-4 and IL-13 induce JAK-dependent tyrosine phosphorylation of the receptor and subsequent STAT6 phosphorylation. Phosphorylated STAT6 dimerizes and translocates to the nucleus where it exerts its canonical functions regulating the transcription of IL-4 target genes [11]. The influence of STAT6 on immune cell regulation and its association with different types of malignancies, including colorectal cancer, has been already investigated [12–17]. In an IL-4-dependent manner Stat6 has been suggested to promote colorectal cancer due to its ability to polarize myeloid cells in a wound–healing/tumor-promoting alternative phenotype, which differentiates these cells from the tumor-suppressing IFNγ-mediated classical activation phenotype [18]. However, whether Stat6 in intestinal epithelial cells can also directly contribute to colon carcinogenesis has not been fully addressed yet.

Using genetically modified mice and different well-established cancer models we show that Stat6 affects chromatin condensation in intestinal epithelial cells thereby interfering with alkylation-induced DNA-damage response and p53 activation independently of its canonical IL-4 signaling functions. Consequently, Stat6 suppresses colitis-associated tumorigenesis, protecting the intestinal epithelium from apoptosis and severe tissue damage. In stark contrast, in a model of sporadic inflammation-independent carcinogenesis this Stat6 controlled mechanism supports tumor promotion highlighting a so far unrecognized nuclear function of unconventionally activated Stat6 in IEC.

## Results

### Stat6 suppresses colitis-associated tumorigenesis

**Fig. 1:**
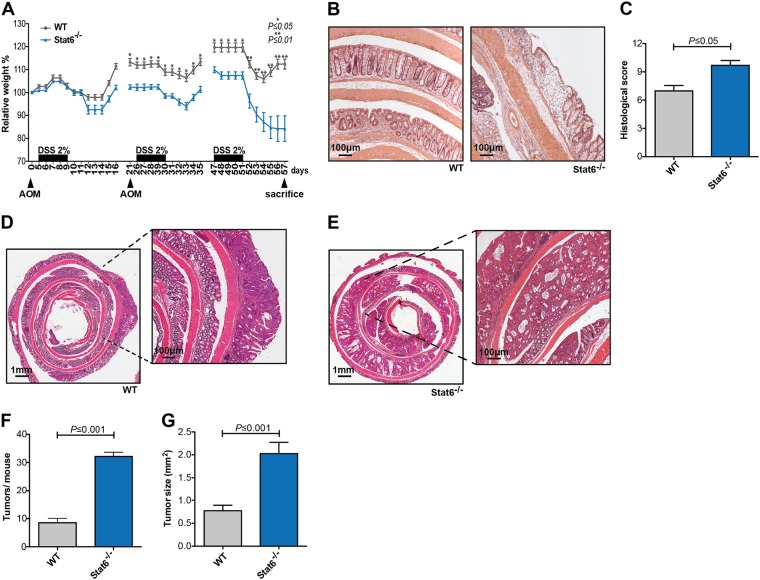
*Stat6* suppress colitis-associated carcinogenesis **a**–**g**. **a**–**g** Colitis-associated carcinogenesis (CAC) model performed with WT and *Stat6*^−/−^ mice (*n* = 8/genotype); **b**, **c** H&E stained sections of colons and quantification of histological damage after chronic colitis in WT and *Stat6*^−/−^ mice (WT *n* = 5; *Stat6*^−/−^ *n* = 7); **d**, **e** Representative H&E stained sections of colonic tumors from WT and *Stat6*^−/−^ mice; **f**, **g** Histological analysis and quantification of tumor incidence and tumor growth in WT and *Stat6*^−/−^ mice at the end of the CAC model (*n* = 8/genotype). Data are mean ± SEM

To determine Stat6 function during colitis-associated carcinogenesis, we subjected animals from both genotypes to the well-established AOM/DSS model [19, 20]. Loss of *Stat6* rendered mice very susceptible to chronic colitis which caused significantly more weight loss in *Stat6*^−/−^ mice compared to control mice after the second DSS cycle (Fig. 1a). Weight loss became even more pronounced in *Stat6*-deficient mice after the third cycle requiring early termination of the model on day 57. In line with a more severe form of colitis, histological analysis revealed increased tissue damage and epithelial ulceration in *Stat6*^−/−^ mice (Fig. 1b, c). Moreover, the number as well as the size of colonic tumors was significantly higher in *Stat6*^*−/−*^ mice (Fig. 1d–g). Tumors from both genotypes were located exclusively in the colon and exhibited classical tubular morphology with moderate dysplasia.

**Fig. 2:**
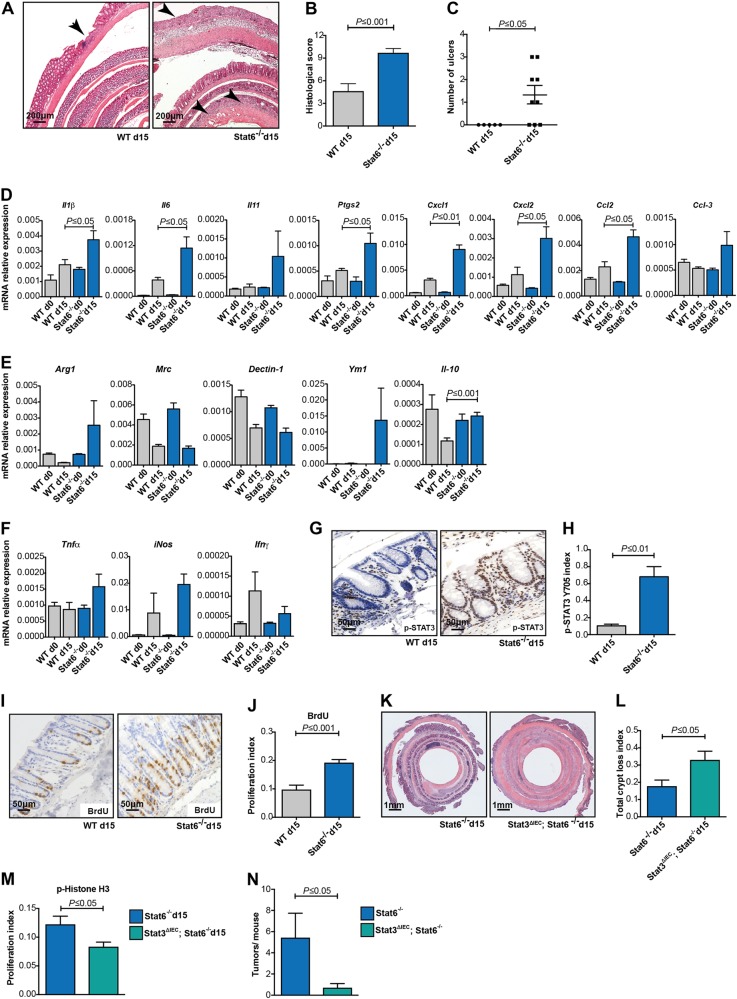
*Stat6* deletion increases DSS-induced colitis severity **a**–**n**. **a**–**m** The mice were subjected to one i.p. injection of AOM (day 0) and exposed to 2% DSS in the drink water for five days (days 5–10), being killed after 5 days of recovery in normal water (day 15). **a** Representative H&E stained sections of colons from WT and *Stat6*^−/−^ mice (H&E); **b**, **c** Histological scoring of mucosal damage, inflammation and ulceration in WT and *Stat6*^−/−^ mice at day 15 of the AOM/DSS model (WT *n* = 5; *Stat6*^−/−^ *n* = 9); **d**–**f** RT-PCR, expression analysis of inflammation-related and M2/M1 genes in the mucosa of unchallenged (day 0) and AOM/DSS-treated mice at day 15 (WT d0/*Stat6*^−/−^ d0 *n* = 3; WT d15/*Stat6*^−/−^ d15 *n* = 5); **g**, **h** Immunohistochemical analysis and quantification of p-STAT3 Y705 performed with colonic sections of WT and *Stat6*^−/−^ mice (WT d15 *n* = 5; *Stat6*^−/−^ d15 *n* = 4); **i**, **j** Immunohistochemical analysis and quantification for BrdU incorporation in WT and *Stat6*^−/−^ mice (WT d15 *n* = 5; *Stat6*^−/−^ d15 *n* = 9); **k** Representative H&E stained sections from colons of *Stat6*^−/−^ and *Stat3*^*Δ*IEC^; *Stat6*^−/−^ double knockout mice at day 15 of AOM/DSS model; **l** Quantification of areas of total crypt loss *in Stat6*^−/−^ and *Stat3*^*Δ*IEC^; *Stat6*^−/−^ double knockout at day 15 of AOM/DSS model (*Stat6*^−/−^ d15 *n* = 16; *Stat3*^*Δ*IEC^; *Stat6*^−/−^ d15 *n* = 15); **m** Index of p-Histone H3 expressing IEC in *Stat6*^−/−^ and *Stat3*^*Δ*IEC^; *Stat6*^−/−^ double knockout mice at day 15 of the AOM/DSS model (*Stat6*^−/−^ d15 *n* = 5; *Stat3*^*Δ*IEC^; *Stat6*^−/−^ d15 *n* = 10); **n** Tumor incidence in *Stat6*^−/−^ (*n* = 7) and *Stat3*^*Δ*IEC^; *Stat6*^−/−^ mice (*n* = 10) at the end of the CAC model. Animals were exposed to reduced DSS concentration during the CAC cycles to prevent mortality (0.5%, 0.8 and 1% DSS, respectively). Data are mean ± SEM

To directly confirm increased susceptibility to DSS-induced colitis, we examined the acute inflammatory response in WT and *Stat6*^−/−^ mice after the first cycle of DSS on day 15 of the colitis-associated carcinogenesis (CAC) model. Indeed, *Stat6* deletion led to more pronounced tissue damage (Fig. 2a–c) and increased expression of genes encoding pro-inflammatory cytokines *IL-1β*, *Ptgs2*, *IL-6*, and *IL-11* as well of the chemo-attractants *Cxcl1*, *Cxcl2*, *Ccl3*, and *Ccl2* in colonic mucosa (Fig. 2d). Considering Stat6 the main transcription factor downstream of IL-4 receptor engagement, which has been associated with M2 macrophage polarization [21], we aimed to evaluate in *Stat6*-deficient inflamed mucosa the expression of genes typically expressed upon M2 polarization. Surprisingly, real-time PCR analysis did not reveal any significant reduction in *Arg1*, *Mrc*, Dectin-1, *Ym1* or *Il10* expression in DSS-challenged *Stat6*^−/−^ mice compared to WT animals (Fig. 2e). Moreover, only moderate yet non-significant differences were found in the expression of M1-associated genes, such as *Tnf*α*, iNos, Ifnγ* as well as IFNγ-regulated genes between the two genotypes on day 15 of the AOM/DSS regimen (Fig. 2f and Figure Supp. 1A). Importantly, however, in agreement with elevated *Il6* and *Il11* gene expression in *Stat6*-deficient DSS-challenged mucosa, tyrosine-phosphorylated STAT3 (Y705) was markedly enhanced in *Stat6*^−/−^ IEC (Fig. 2g, h and Figure Supp. 1B). Previously, we had reported that epithelial regeneration during acute colitis was dependent on Stat3 activation in IEC [22]. In line with this notion, *Stat6*^−/−^ IEC proliferated significantly more during the healing phase of acute colitis (Fig. 2i, j). Importantly, also *Stat6*-deficient tumor epithelia were characterized by increased Stat3 phosphorylation suggesting that enhanced gp130-dependent Stat3 activation triggered by IL-6 and IL-11 could account for the pro-proliferative, pro-tumorigenic phenotype of *Stat6*^−/−^ mice (data not shown). To confirm this, we generated *Stat3*^*Δ*IEC^; *Stat6*^−/−^ double mutants and analyzed IEC proliferation on day 15 of the AOM/DSS model. Expectedly, absence of Stat3 reduced proliferation in *Stat6*-deficient IEC, which led to even further impairment of epithelial regeneration and massive crypt loss when compared to *Stat6*-single mutants (Fig. 2k–m). Consequently, *Stat3*-deficiency in IEC reduced tumor growth in *Stat6*^−/−^ mice (Fig. 2n).

### Stat6 expression in IEC prevents inflammation-associated tumorigenesis

**Fig. 3:**
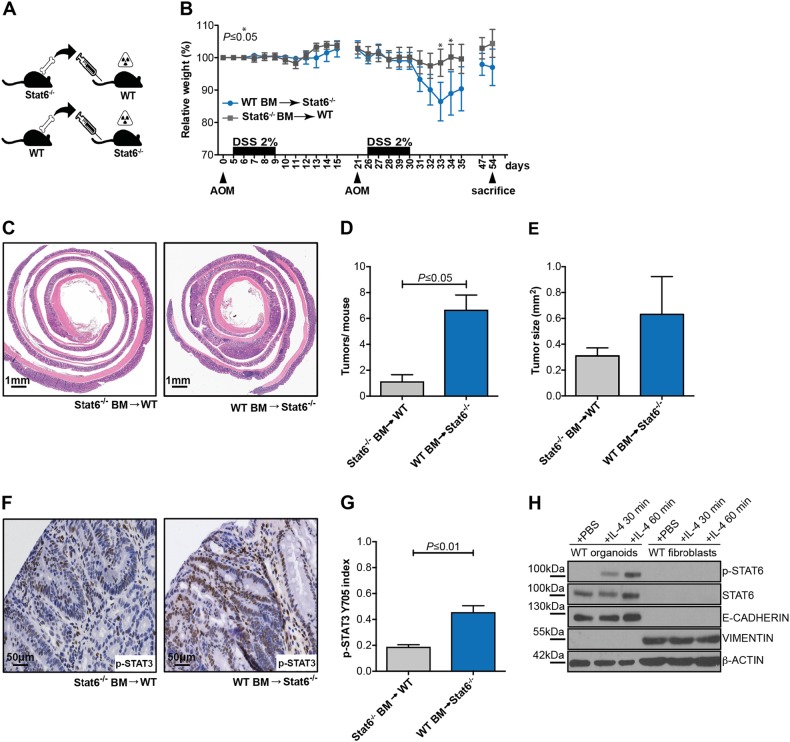
*Stat6* expression in intestinal epithelial cells prevents inflammation-associated carcinogenesis **a**–**h**. **a** *Stat6*^*−/−*^BM > WT or WT BM > *Stat6*^*−/−*^ reconstituted mice were challenged with the CAC model; **b** Weight changes of transplanted mice during AOM/DSS model; **c** Representative H&E stained sections of *Stat6*^*−/−*^BM > WT and WT BM > *Stat6*^*−/−*^ mice colonic tumors; **d**, **e** Tumor incidence and tumor size in *Stat6*^*−/−*^BM > WT and WT BM > *Stat6*^*−/−*^ mice at the end of the CAC model (*Stat6*^*−/−*^BM > WT *n* = 7; WT BM > *Stat6*^*−/−*^ *n* = 6); **f**, **g** Immunohistochemical analysis and quantification of p-STAT3 Y705 in tumors from *Stat6*^*−/−*^BM > WT and WT BM > *Stat6*^*−/−*^ mice (*n* = 3/genotype); **h** Immunoblot analysis of p-STAT6 Y641 from WT colon organoids and fibroblast lysates stimulated with recombinant mouse IL-4 for 30 and 60 min. E-cadherin and Vimentin antibodies were used to ensure the respective epithelial and mesenchymal origin of the samples. β-actin as loading control. Data are mean ± SEM

To determine whether the increased sensitivity to DSS-induced inflammation and elevated tumor load in *Stat6*^−/−^ mice was dependent on loss of Stat6 in hematopoietic cells or another cell compartment, we performed adoptive transfer experiments. To this end, we transplanted *Stat6*^−/−^ bone marrow into lethally irradiated WT mice (*Stat6*^*−/−*^BM > WT) or conversely transferred WT bone marrow into *Stat6*^−/−^ recipients (WT BM > *Stat6*^*−/−*^) (Fig. 3a). Six weeks after transplantation when mice were successfully reconstituted (Figure Suppl. 1C), animals were challenged with AOM/DSS and tumor growth was assessed. Similarly to non-transplanted *Stat6*^−/−^ mice, *Stat6*^*−/−*^ animals receiving WT bone marrow were very susceptible to DSS-induced colitis and because of their increased weight loss and rectal bleeding mice had to be euthanized on day 54 of the model (Fig. 3b). Tumor incidence, tumor size as well as STAT3 phosphorylation was increased in *Stat6*^−/−^ mice that had received WT bone marrow (Fig. 3c–g), indicating that the lack of Stat6 expression in non-hematopoietic cells such as IEC or stromal fibroblasts was responsible for the elevated tumor load. Because Stat6 activation was only detectable in intestinal organoids but not intestinal fibroblasts (Fig. 3h), we reasoned that Stat6 expression in IEC most likely conferred tumor suppression in this model.

### Stat6 loss reduces alkylation-induced DNA damage, due to changes in the expression of chromatin-remodeling proteins

**Fig. 4:**
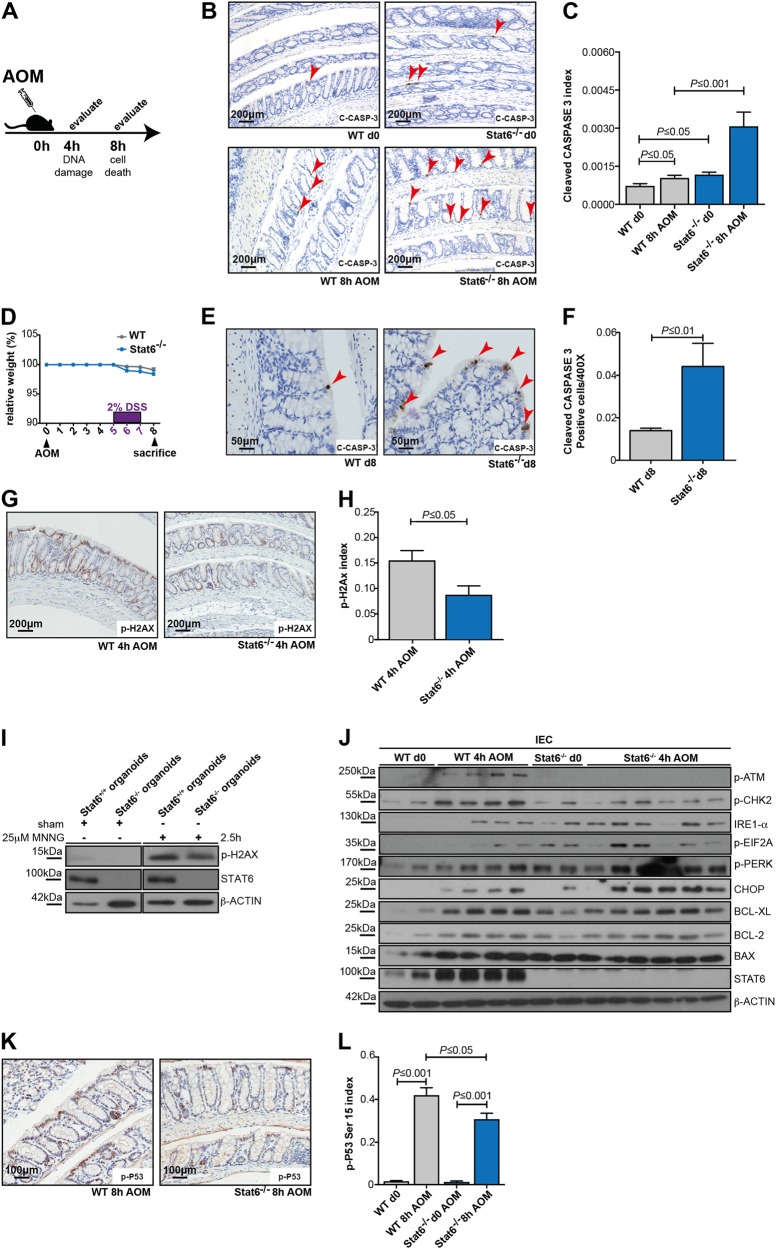
Stat6 regulates IEC death and alkylating-inducing DNA damage **a**–**l**. **a**–**c** Immunohistochemical staining and index of cleaved CASPASE 3 expression in unchallenged (d0) and AOM-treated (8 h) WT and *Stat6*^*−/−*^ IEC (WT d0 *n* = 6; *Stat6*^−/−^ d0 *n* = 3; WT 8 h AOM *n* = 8; *Stat6*^−/−^ 8 h AOM *n* = 6); **d**–**f** Immunohistochemical staining and index of cleaved CASPASE 3 expression in IEC on day 8 (d 8) of the CAC model (*n* = 5/genotype); **g**, **h** Immunohistochemical staining and index of p-H2AX in AOM-treated (4 h) WT and *Stat6*^*−/−*^ IEC (WT 4 h AOM *n* = 7; *Stat6*^−/−^ 4 h AOM *n* = 6); **i** Immunoblot analysis of H2AX phosphorylation and STAT6 in WT (*Stat6*^*+/+*^) and *Stat6*^*−/−*^ colonic organoids treated 2.5 h with 25 μM MNNG or 0.03% DMSO in PBS (sham). β-actin as loading control; **j** Immunoblot analysis of p-ATM, p-CHK2, IRE1-a, p-EIF2A, p-PERK, CHOP, BCL-XL, BCL-2, BAX, and STAT6 performed with IEC lysates from unchallenged (d0) or AOM-treated (4 h) mice. β-actin as loading control; **k**, **l** Immunohistochemical staining and index of p-P53 S15 expression in unchallenged (d0) and AOM-treated (8 h) WT and *Stat6*^*−/−*^ IEC (WT d0 *n* = 6; *Stat6*^−/−^ d0 *n* = 3; WT 8 h AOM *n* = 9; *Stat6*^−/−^ 8 h AOM *n* = 6). Data are mean ± SEM

The extent of initial epithelial cell death during the AOM/DSS model substantially determines the outcome of DSS-induced colitis since increased colonic epithelial cell loss causes a more pronounced barrier defect and subsequently a stronger inflammatory response [19, 22, 23]. Considering the results of the adoptive transfer experiments that pointed to a role of Stat6 in epithelial cells in the AOM/DSS model, we hypothesized that Stat6 deletion would affect cell death of colonic epithelia. AOM administration causes DNA alkylation and formation of O6-methylguanine. This triggers activation of DNA damage response pathways culminating in DNA repair as well as apoptosis [24, 25]. Indeed, the number of apoptotic IEC was markedly enhanced in *Stat6*^−/−^ mice within 8 h after AOM administration (Fig. 4a–c). Moreover, also in response to DSS *Stat6*-deficient epithelia expressed more cleaved caspase 3 positive cells on day 8 of the AOM/DSS model (Fig. 4d–f) supporting the notion that the increased inflammatory response observed in *Stat6*^−/−^ mice during colitis was a consequence of enhanced epithelial cell loss. To determine how Stat6 affected AOM-induced apoptosis, we first examined the phosphorylation of the histone variant H2AX, which comprises one of the earliest events in response to DNA damage [26]. Surprisingly, H2AX phosphorylation was reduced in AOM-challenged *Stat6*^−/−^ IEC (Fig. 4g, h). Similarly, H2AX phosphorylation was reduced in colonic organoids challenged with the alkylating agent *N*‐methyl‐*N*′‐nitro‐*N*‐nitrosoguanidine (MNNG) (Fig. 4i), confirming a cell autonomous effect. Yet, in agreement with a reduced DNA damage response in *Stat6*^−/−^ cells ATM and CHK2 phosphorylation was diminished, which was accordingly associated with impaired p53 phosphorylation (Fig. 4j-l). This suggested that the increased cell death in *Stat6*^−/−^ mice was not dependent on p53. Moreover, no difference in expression of BCL-XL, BCL-2 or BAX could be detected between the genotypes in response to the AOM challenge. Instead, we observed enhanced IRE1-α, p-PERK, p-EIF2A, and CHOP expression in *Stat6*^−/−^ IEC indicating an enhanced unfolded protein response (UPR), which could account for apoptosis induction (Fig. 4j) [27, 28].

**Fig. 5:**
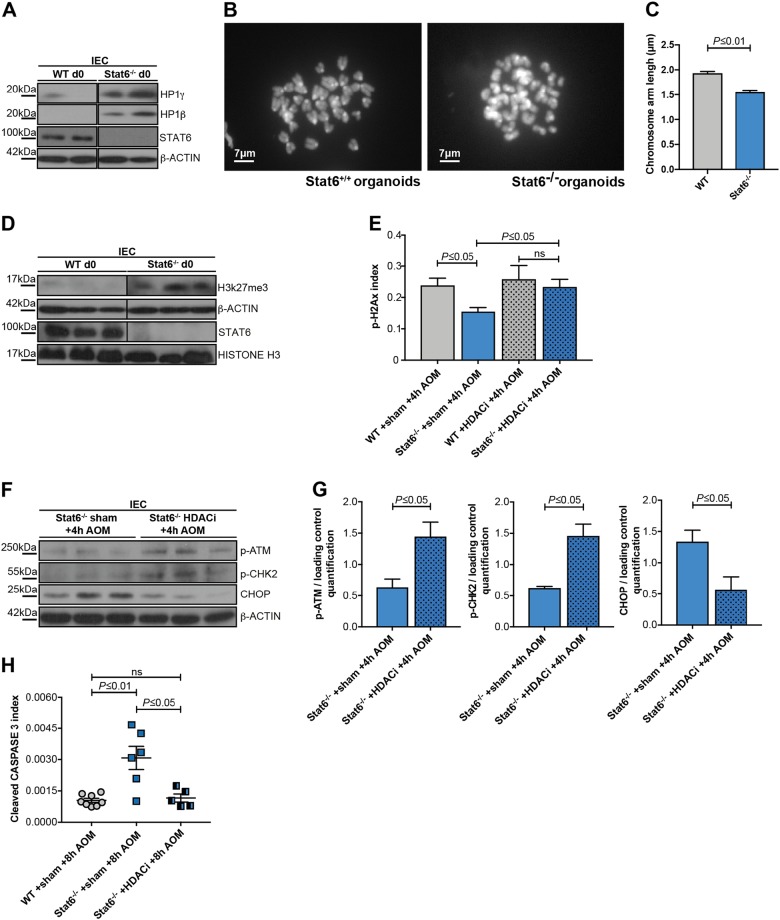
*Stat6* loss affects the expression of chromatin-remodeling proteins and chromatin compaction **a**–**h**. **a** Immunoblot analysis of HP1γ/β in unchallenged (d0) WT and *Stat6*^*−/−*^ IEC. β-actin as loading control. **b**, **c** Karyotype analysis and chromosome arm length measurement in WT and *Stat6*^*−/−*^ colon organoids; **d** Immunoblot analysis of H3K27me3, STAT6, and HISTONE H3 in unchallenged (d0) WT and *Stat6*^*−/−*^ IEC. β-actin as loading control. **e** p-H2AX index in AOM-treated (4 h) WT and *Stat6*^*−/−*^ IEC pretreated with NaCl 0.9% (sham) or HDAC inhibitor (HDACi) for 5 days (WT or *Stat6*^−/−^ + sham + 4 h AOM *n* = 3, respectively; WT or *Stat6*^−/−^ + HDACi + 4 h AOM *n* = 4, respectively); **f**, **g** Immunoblot analysis and quantification of p-ATM, p-CHK2 and CHOP in *Stat6*^*−/−*^ IEC from mice that had been pretreated with NaCl 0.9% (sham) or HDACi for five consecutive days. β-actin as loading control. Results were normalized to the loading control. **h** Index of cleaved CASPASE 3 expression in AOM-treated (8 h) WT and *Stat6*^*−/−*^ IEC from mice that had been pretreated with NaCl 0.9% (sham) or HDACi for five consecutive days (WT + sham + 8 h AOM *n* = 8; *Stat6*^−/−^ + sham + 8 h AOM *n* = 6; *Stat6*^−/−^ + HDACi + 8 h AOM *n* = 5). Data are mean ± SEM

Modifications in heterochromatin structure can affect the DNA-damage response [29–33] and changes in the expression of chromatin-remodeling proteins, such as the heterochromatin protein 1 family members (HP1α/β/γ) may increase sensitivity to DNA damage [34], while STATs can interfere with the expression of HP1 family members [35–37]. Intriguingly, we detected increased levels of HP1 γ and HP1β in the lysates of unchallenged IEC from *Stat6*^*−/−*^ mice as well as in untreated *Stat6*-deficient organoids (Fig. 5a, and Figure Supp. 2A, B). Consequently, karyotyping analysis revealed that *Stat6*-deficient cells exhibited increased chromosome condensation when compared with their WT controls (Fig. 5b, c). In line with impaired chromatin accessibility due to chromosome condensation, expression of the repressive histone mark H3K27me3 was markedly elevated (Fig. 5d). To further validate these results, we pretreated WT and *Stat6*^−/−^ mice for five consecutive days with valproate (valproic acid, 200 mg/kg, daily), a well-known histone deacetylase (HDAC) inhibitor that leads also to chromatin decondensation [38, 39], and evaluated its effect on DNA damage response and IEC apoptosis upon the AOM challenge. Notably, valproate did not only normalize histone H2AX phosphorylation in *Stat6*-deficient IEC comparable to WT levels (Fig. 5e), but also increased ATM and CHK2 phosphorylation (Fig. 5f–g). Moreover, valproic acid decreased CHOP expression and prevented the enhanced IEC apoptosis observed after 8 h in AOM-challenged *Stat6*^−/−^ mice (Fig. 5f, h).

Collectively, these results suggested that increased heterochromatin stability and higher chromatin compaction were responsible for the impaired DNA damage response and increased apoptosis in *Stat6*^−/−^ IEC.

### IL-4Rα in IEC is not involved in Stat6 activation during DNA damage

**Fig. 6:**
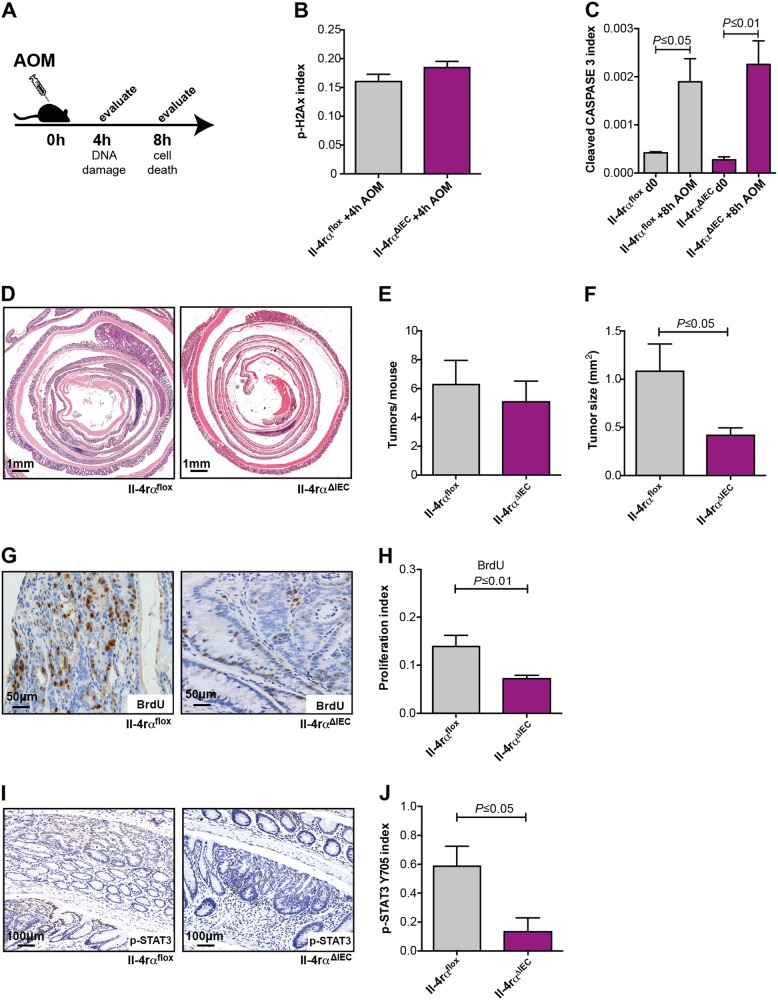
IL-4Rα is not involved in Stat6 activation during DNA damage **a**–**j**. **a**, **b** p-H2AX index in AOM-treated (4 h) *Il-4rα*^*flox*^ (*n* = 5) and *Il-4rα*^*Δ*IEC^ mice (*n* = 6); **a**, **c** Index of cleaved CASPASE 3 expressing IEC in unchallenged (d0) and AOM-treated (8 h) *Il-4rα*^*flox*^ and *Il-4rα*^*Δ*IEC^ mice (*Il-4rα*^*flox*^ d0 *n* = 3; *Il-4rα*^*Δ*IEC^ d0 *n* = 5; *Il-4rα*^*flox*^ + 8 h AOM *n* = 4; *Il-4rα*^*Δ*IEC^ + 8 h AOM *n* = 4); **d**–**j** CAC model performed with *Il-4rα*^*flox*^ and *Il-4rα*^*Δ*IEC^ mice (*n* = 10/genotype); **d** Representative H&E stained sections of colonic tumors from *Il-4rα*^*flox*^ and *Il-4rα*^*Δ*IEC^ mice; **e**, **f** Tumor incidence and tumor size of *Il-4rα*^*flox*^ and *Il-4rα*^*Δ*IEC^ mice at the end of the CAC model (*n* = 10/genotype); **g**, **h** BrdU incorporation in tumors from *Il-4rα*^*flox*^ (*n* = 5) and *Il-4rα*^*Δ*IEC^ (*n* = 7) mice; **i**, **j** Immunohistochemical staining and index of p-STAT3 Y705 expressing tumor epithelia in *Il-4rα*^*flox*^ and *Il-4rα*^*Δ*IEC^ mice (*n* = 3/genotype). Data are mean ± SEM

To examine whether the Stat6-dependent effects on DNA damage were controlled by IL-4 receptor engagement in IEC, we generated *Il-4rα*^*Δ*IEC^ mice and examined their sensitivity to AOM injection as well as tumor incidence in the AOM/DSS model. In contrast to *Stat6*^−/−^ mice, IEC-restricted loss of IL-4Rα did not reduce H2AX phosphorylation or affected mutagen-induced cell death after AOM injection (Fig. 6a–c). Similarly, tumor incidence was not affected in *Il-4rα*^*Δ*IEC^ mice in the AOM/DSS model (Fig. 6d, e). However, tumors were significantly smaller which correlated with a reduced BrdU incorporation and reduced Stat3 activation in tumor epithelia (Fig. 6d, f–j). Thus, Stat6 controls the DNA damage response independently of IL-4Rα dependent signaling in IEC.

### Stat6 promotes sporadic inflammation-independent carcinogenesis

**Fig. 7:**
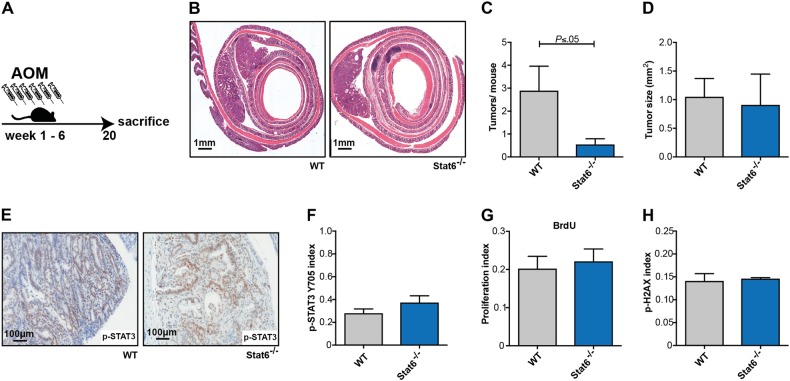
Stat6 promotes sporadic inflammation-independent carcinogenesis **a**–**h**. **a** Colitis-independent carcinogenesis (sporadic) model performed with WT and *Stat6*^*−/−*^ mice; **b** Representative H&E stained sections of colons from WT and *Stat6*^−/−^ mice 20 weeks after the first AOM injection; **c**, **d** Tumor incidence and tumor size in WT and *Stat6*^−/−^ mice (WT *n* = 9; *Stat6*^−/−^ *n* = 11); **e**, **f** Immunohistochemical staining and index of p-STAT3 Y705 expressing tumor epithelia in WT and *Stat6*^−/−^ mice (WT *n* = 5; *Stat6*^−/−^ *n* = 3); **g** BrdU incorporation in tumors from WT and *Stat6*^−/−^ mice (WT *n* = 4; *Stat6*^−/−^ *n* = 3); **h** Index of p-H2AX expressing tumor epithelia in WT (*n* = 5) and *Stat6*^−/−^ mice (*n* = 3)

Our data suggested that due to increased initial IEC death a more severe inflammatory response during the DSS-induced colitis led to an upregulation of Stat3-activating cytokines, which promoted tumor growth and compensated the loss of initiated epithelial cells. Therefore, we hypothesized that *Stat6*^−/−^ mice should be protected from AOM-induced tumorigenesis when this model was done in the absence of DSS-induced colitis. Indeed, repetitive AOM-injections (six weekly i.p. injections) represent a valuable model for sporadic tumorigenesis, which leads to formation of colonic tumors within 20 weeks (Fig. 7a). As expected, increased apoptotic cell death in response to AOM led to a reduction in tumor number in this model (Figs. 4c and 7b, c). In line with unchanged Stat3 activation in tumor epithelia, tumor sizes and proliferation rates determined by BrdU incorporation were comparable to control mice (Fig. 7d–g). Interestingly, also H2AX phosphorylation was not decreased in established *Stat6*-deficient tumors (Fig. 7h). Collectively, these data clearly support a tumor promoting function of Stat6 in IECs during early sporadic colorectal carcinogenesis.

## Discussion

A considerable amount of data endorses the concept that Stat6 is critical to mediate transcriptional activation upon IL-4 and IL-13 stimulation, emphasizing the important canonical IL-4/IL-13 dependent functions of Stat6 in immune cells [11–13]. However, Stat6 direct functions on intestinal epithelial cells during homeostasis and colorectal carcinogenesis remained elusive. Here, using genetically modified mice and different experimental models of colorectal carcinogenesis we show that Stat6 unexpectedly exerts important direct IL-4Rα independent cell-intrinsic functions in IEC.

Intestinal homeostasis is maintained due to the close interaction of many different factors, including gut microbiota composition, immune system activation and intestinal epithelial cell differentiation and survival. The balance between these factors is crucial to the host, and its disruption can lead to the development of a number of diseases. The finding that *Stat6* loss can directly regulate IEC death in vivo, and therefore affect colitis severity and colorectal carcinogenesis is important to clarify its function during these two different conditions. During DSS-induced colitis *Stat6* loss triggers enhanced IEC apoptosis, causing intense tissue damage and massive inflammatory response, with secretion of many pro-inflammatory chemokines and cytokines that later activate intestinal epithelial cell-intrinsic survival mechanisms. The enhanced expression of *Il-6* and *Il-11*, followed by strong Stat3 activation and enhanced BrdU incorporation found in the mucosa of *Stat6*^*−/−*^ mice at day 15 of the CAC model support this notion. Stat3-overexpressing enterocytes unequivocally disturb the regeneration of the damaged epithelium, inducing aberrant IEC proliferation and later tumorigenesis [22]. The increased tumorigenesis found in these mice at the end of the CAC model along with the results obtained with *Stat3*^*Δ*IEC^; *Stat6*^−/−^ mice support the concept that tumor formation and growth in *Stat6*-deficient mice is promoted by Stat3 activation in enterocytes at the earlier DSS-induced colitis. Unexpectedly, gene expression analysis of the colonic mucosa of *Stat6*^*−/−*^ mice during colitis as well as of tumor samples collected at the end of the CAC model did not detect significant changes in the expression of alternative and classical activation genes, suggesting that these markers might not play a critical role in this experimental model (Fig. 2e, f and data not shown). Recently, Leon-Cabrera et al., also reported that *Stat6-*deficient mice exhibit increased IEC cell death at the early stages of CAC [40]. Yet, in this study *Stat6*^*−/−*^ mice developed a reduced inflammatory response and a lower number of colonic tumors, possibly due to a different experimental set up (i.e. lack of cohoused control mice or no exchange of bedding) causing changes in the intestinal microbiome. While our finding that *Stat6*^*−/−*^ mice are more susceptible to mucosal damage is in agreement with other previous reports [41, 42], our results markedly differ. Previous studies suggested that *Stat6*^−/−^ mice enhanced susceptibility mucosal damage is due to changes in the phenotype of myeloid cells, especially macrophages. However, in these reports the immediate effects of *Stat6* deletion on intestinal epithelial cell death and survival had not been examined.

By now it is widely accepted that a pro-inflammatory intestinal microenvironment predisposes to the activation of oncogenes, which later trigger the expression of tumor-promoting molecules driving neoplastic transformation [3, 25, 43–50]. We had previously demonstrated that upon genotoxic stress, the early death of the intestinal initiated epithelium leads to higher protection against tumor formation [22, 25]. This effect is based on the elimination of genetically damaged epithelial cells, which when allowed proliferating would acquire and accumulate additional mutations that would provide them with cell-autonomous growth advantages. As *Stat6*-deficient IEC were more susceptible to undergo apoptosis upon AOM challenge it was expected that during sporadic colitis-independent carcinogenesis Stat6^−/−^ mice would exhibit reduced number of tumors. Surprisingly, we found that AOM-induced apoptosis appears to be independent of p53 activation in these mice, which was further confirmed by crossing *Stat6*^−/−^ mice to intestinal cell-specific deleted *Tp53* mice (*Tp53*^ΔIEC^). The resulting animals were challenged with sporadic colitis-independent cancer model, and similarly to AOM-induced *Stat6*^−/−^ mice, *Stat6*^−/−^;*Tp53*^ΔIEC^ double mutants developed a significantly lower number of tumors (De Oliveira, unpublished observations), confirming the essential role of early carcinogen-triggered apoptosis in this model. Supporting our findings on sporadic carcinogenesis, it has been recently shown that *Stat6* deletion in the *Apc*^Min/+^ mouse model reduced the incidence of polyps in the small intestine, and that *Stat6*-deficient *Apc*^Min/+^ polyps exhibited increased cell death [18]. However, this effect was attributed to increased cytotoxic activity of *Stat6*-deficient CD8^+^ T cells.

Stats, as well as Janus Kinases (Jaks), are best known by their canonical functions which upon phosphorylation mediate intracellular signals derived from cytokines to control transcriptional activation of several different target genes. However, Stats have less-well described kinase-independent non-canonical functions [51]. Nuclear unphosphorylated STAT92E has been found to influence heterochromatin stability in *Drosophila melanogaster* due to its ability to associate with HP1, an important chromatin-remodeling protein. In this species, STAT92E phosphorylation reduces its heterochromatin association, leading to HP1 displacement and heterochromatin destabilization [37]. Additionally, decreased levels of unphosphorylated STAT92E—thus reducing heterochromatin stability—induce low resistance to DNA damage during genotoxic stress conditions [31]. Although conversely to what was observed in *Drosophila*, the finding that *Stat6*-deficient enterocytes are more resistant to AOM-induced DNA damage is supported by elevated expression of HP1 proteins as well as the heterochromatin repressor mark H3K27me3 in these cells. HP1 strong expression contributes directly to the chromatin compaction, providing extra protection against chromosomal breakages [31], whereas H3K27me3 increased expression confirms its condensed repressed status [52]. HDAC inhibitor treatment of *Stat6*^−/−^ mice causing chromatin decondensation validated these observations. Nevertheless, the exact regulatory mechanisms underlying changes in chromatin structure and UPR in *Stat6*-deficient enterocytes and whether STAT6 can direct physically interact with HP1 proteins remain to be established.

To date, Stat6 function during tumorigenesis has been mostly defined in the context of IL-4/IL-13 engagement in inflammatory immune cells. Our finding that in IEC Stat6 is involved in chromatin condensation in an IL-4Rα-independent manner may also impact cytotoxic therapy and suggests that targeting IL-4/IL-13 or Stat6 might have a very different therapeutic outcome.

## Materials and methods

### Animal experiments

Stat6 knockout (Stat6^−/−^) and C57BL/6 (WT) mice were both from the Jackson Laboratory, Bar Harbor, ME. *Il-4rα*^*Δ*IEC^ and *Stat3*^*Δ*IEC^ mouse strains were obtained by crossing *Il-4rα* [53] or *Stat3*^*flox*^^54^ to *villin*-cre mice [55]. *Stat3*^*Δ*IEC^; *Stat6*^−/−^ double knockout mice were generated by further crossing *Stat3*^*Δ*IEC^ to *Stat6*^*−/−*^ mice. *Il-4rα*^*Δ*IEC^ and *Stat3*^*Δ*IEC^; *Stat6*^−/−^ mice were kept on a mixed background. All animals were bred and housed at the Georg-Speyer-Haus animal facility under specific pathogen-free conditions and controlled 12-hours light/dark cycle. Gender and age-matched mice (ranging from six to 12 weeks old) were used in all experiments. To ensure a balanced microbiome between genotypes, cage beddings were mixed twice a week between genotypes, for a minimum of 2 weeks, prior to all experiments. Additionally, animals kept under long experimental periods (e.g. CAC or sporadic carcinogenesis model) had their bedding mixed weekly. Littermates were used as controls in experiments performed with the *Il-4rα*^*Δ*IEC^ and *Stat3*^*Δ*IEC^; *Stat6*^−/−^ mice. Food and water were provided ad libitum. Experimental colitis was induced by administrating 2% dextran sodium sulphate (DSS) (36–50 kDa, MP Biomedicals, CA) in drinking water for 5 days, followed by five additional days of regular drinking water. Severity of colitis was histologically evaluated as previously described [56]. The total crypt loss index was calculated measuring the length of total crypt loss areas (in μm) and dividing it by the total length of the colonic segment. Experimental colitis-associated carcinogenesis (CAC) was performed as previously described [43] using 10 mg/kg AOM (Sigma-Aldrich, St. Louis, MO) and 2% DSS, unless indicated otherwise. Experimental colitis-*independent* (sporadic) carcinogenesis was performed subjecting animals weekly, for 6 weeks, to i.p. injections of AOM (10 mg/kg). Mice were killed 20 weeks after the first AOM injection. To evaluate the acute effects of AOM, animals were sacrificed 4 or 8 h after AOM (10 mg/kg) injection. Adoptive transfer experiments were performed subjecting mice to a whole-body gamma-radiation of 9.5 Gy. Bone marrow from donor mice was collected from femoral bones and cell number determined. Approximately 1 × 10^6^ cells were injected into the tail vein of lethally irradiated mice. Transplanted mice were given antibiotic water (2 mg/ml Ciprobay, Bayer, Leverkusen, NW) for 14 days, followed by 14 days of regular water before being subjected to CAC. Animals were monitored and weights recorded daily during the colitis-associated experiments and three times per week in colitis-independent experiments. In all animal experiments mice with weight loss more than 20% of their initial body weight were euthanized. Evaluators were blinded to the group information and animal data analyses presented are representative of two or more experiments. All procedures were reviewed and approved by the Regierungspräsidium Darmstadt, Germany.

### Intestinal epithelial cell proliferation assessment

Mice were i.p. injected with 75 mg/kg of BrdU (Sigma-Aldrich), 90 min before killing. IEC proliferation was determined by staining colonic paraffin tissues sections with anti-BrdU antibody (Serotec, Raleigh, NC). Stained sections were scanned using Aperio Scanscope XT and the proliferation index was calculated dividing the number of BrdU positive epithelial cells by the total number of epithelial cells, using Aperio Imagescope software (both, Leica Biosystems, Nussloch, BW).

### Immunohistochemistry

Immunohistochemistry was performed as previously described [45]. The following antibodies were used: phospho-STAT3 Y705, phospho-Histone H2AX S139, phospho-Histone H3 S10, and cleaved Caspase 3 (all from Cell Signaling Technology, Danvers, MA). After image acquisition using an Aperio Scanscope XT (Leica), the staining indexes were calculated dividing the number of positive epithelial cells/nuclei by the total number of epithelial cells/nuclei, using Aperio Imagescope software (Leica Biosystems). The resulted ratio is shown as index.

### Immunoblot analysis

Tissues/cell lysate pellets were subjected to SDS-PAGE, transferred into 0.45 μm PVDF membranes and blocked 30 minutes at room temperature with 3% skim milk (Sigma-Aldrich), prior overnight incubation with the following primary antibodies: phospho-ATM S1981 and STAT6 (all from Santa Cruz Biotechnology, Dallas, TX); β-actin (Sigma-Aldrich); Vimentin, p-STAT6 Y641, Histone H3, HP1γ, and H3K27me3 (all from Abcam, Cambridge, UK); BAX, IRE1-α, phospho-EIF2A, phospho-CHK2 T387, CHOP, phospho-STAT3 Y705, and phospho-Histone H2AX S139 (all from Cell Signaling Technology); HP1β (Thermo Fischer Scientific, Waltham, MA); STAT3, BCL-2, BCL-XL, and E-cadherin (BD Biosciences, Franklin Lakes, NJ); phospho-PERK (US Biologicals, Salem, MA). After 30 min incubation with their respectively HRP-conjugated secondary antibodies (GE healthcare, Chicago, IL) membranes were developed using the SuperSignal West Pico Chemoluminescence Substrate (Thermo Fischer Scientific). Western blots were quantified using ImageJ 1.43u Software (National Institutes of Health, Bethesda, Maryland, USA. https://imagej.nih.gov/ij/, 1997–2016).

### Intestinal organoids

Colonic mouse organoids were isolated and kept as previously described [56]. Organoids at passage 2–4 were used for experiments.

### Intestinal epithelial cell isolation

Colons were flushed with ice-cold PBS, cut into small pieces and incubated in 10 ml of pre-warmed (37 °C) HBSS medium supplemented with 5 mM EDTA (Carl Roth, Karlsruhe, BW) and 2 mM DTT (Sigma-Aldrich). Samples were shook (80 rpm) for 10 min at 37 °C. Next, the tissue was vortexed for 30 s and put to rest on ice for more 30 s to allow the *lamina propria* to precipitate. The supernatant was transferred to a new 50 ml conic tube passing through a 70 μm mash and further centrifuged for 5 min at 500 × *g*, 4 °C. The cell pellet resuspended with 3 ml of ice-cold PBS and divided into 1.5 ml Eppendorf tubes. These were further centrifuged for 5 min at 2000 rpm, 4 °C, and the pelleted IEC immediately frozen in liquid nitrogen. Samples were stored at −80 °C until use.

### Karyotype analysis

The karyotyping protocol was kindly provided by Dr. Meritxell Huch (Wellcome Trust/Cancer Research UK, Cambridge, UK). Colon organoid cultures from WT (Stat6^+/+^) and Stat6^−/−^ mice were incubated 24 h with 0.05 μg/ml Colcemid (GIBCO, Calrsbad, CA) in complete ERN medium. Organoids were dissociated into single cells using TrypLE Express (Thermo Fischer Scientific). Single-cells was resuspended in 1 ml 0.075 M KCl, and incubated for 10 min at 37 °C. Next, 1 ml of MeOH:CH_3_OOH solution (3:1) was added and cells centrifuged for 5 min, 1500 rpm, RT. Supernatant was removed, cells were again resuspended in 1 ml of MeOH:CH_3_OH solution (3:1) and incubated for 20 min at RT. After incubation, cells were centrifuged for 5 min, 1500 rpm at RT and stored at −20 °C overnight. Next day, 850 μl of MeOH:CH_3_OOH solution was removed, and cells were resuspended in the remaining volume. The cell suspension was then placed drop-wise on glass slides (SuperFrost Plus, Thermo Fischer Scientific) from a height of ~50 cm, and allowed to air-dry. Finally, slides were covered with ProLong Gold Antifade Mountant with DAPI (Thermo Fischer Scientific) and glass coverslips. Visualization and analysis of mitotic chromosomes compaction was performed measuring the Euclidean distance (in μm) from the centromere to the end of the chromosome arm using Zeiss Imager.M2 microscope and AxionVision SE Rel. 4.9.1 software (Zeiss, Oberkochen Germany).

### RNA analysis

RNA analysis was performed as previously described and quantified by real-time PCR [56]. Primer sequences are available on request.

### Statistical analysis

Data are presented as mean ± SEM. Statistical analysis was performed with Prism7 software (GraphPad Software Inc., La Jolla, CA) using two-tailed Student’s *t*-test, Mann–Whitney test or ANOVA followed by Bonferroni post hoc test. *P* < 0.05 was considered statistically significant.

## Electronic supplementary material

Supplementary Figures 1-2
