# Supplementary material for: Loss of Stat6 affects chromatin condensation in intestinal epithelial cells causing diverse outcome in murine models of inflammation-associated and sporadic colon carcinogenesis
Source: Oncogene. 2018 Oct 23;38(11):1787–801. doi: 10.1038/s41388-018-0551-2 (PMC6756235; doi:10.1038/s41388-018-0551-2)
Supplement: Supplementary file 1 — Supplementary Figures 1-2 [file 41388_2018_551_MOESM1_ESM.pdf]

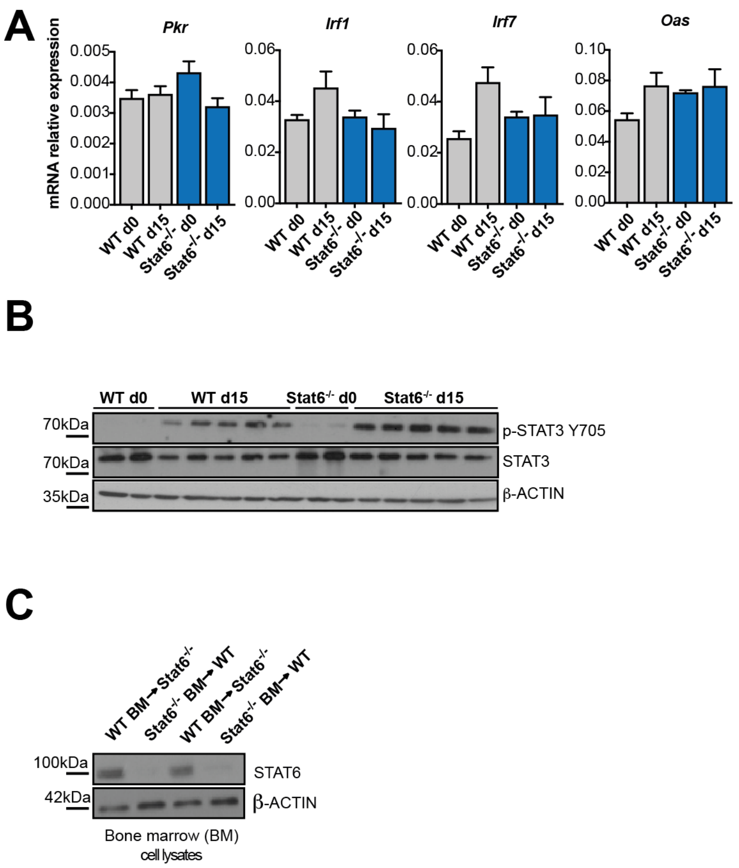


**Supplementary Figure 1 (A-C).** (A) RT-PCR, expression analysis of IFNγ-related genes in the mucosa of unchallenged (day 0) and AOM/DSS-treated WT and *Stat6*$^{-/-}$ mice on day 15 (WT d0/*Stat6*$^{-/-}$ d0 n=3; WT d15/*Stat6*$^{-/-}$ d15 n=5); (B) Immunoblot analysis of p-STAT3 Y705 and STAT3 in unchallenged mucosa (d0) and AOM/DSS-treated (d15) colon from WT and *Stat6*$^{-/-}$ mice. β-ACTIN as loading control; (C) Immunoblot analysis of bone marrow from *Stat6*$^{-/-}$*BM* >WT and WT BM >*Stat6*$^{-/-}$ mice at the end of the CAC model. β-ACTIN as loading control. Data are mean ±SEM.

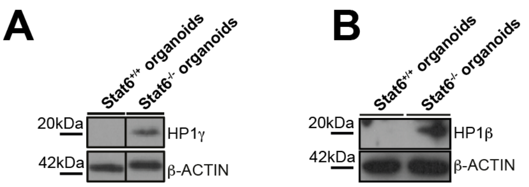


**Supplementary Figure 2 (A-B).** (A-B) Immunoblot analysis of HP1γ and HP1β expression in unchallenged WT and *Stat6*$^{-/-}$ colonic organoids; β-ACTIN as loading control.
